# Supplementary material for: Learning to detect AI texts and learning the limits
Source: PLoS One. 2025 Oct 15;20(10):e0333007. doi: 10.1371/journal.pone.0333007 (PMC12527182; doi:10.1371/journal.pone.0333007)
Supplement: S2 File — All data and scripts used in the study, some additional descriptive statistics, scalable formate figures. (ZIP) [file pone.0333007.s002.tgz › data and scripts/OSF/scripts/analysis/Dependencies/GLS4_distance_vs_Confidence.pdf]

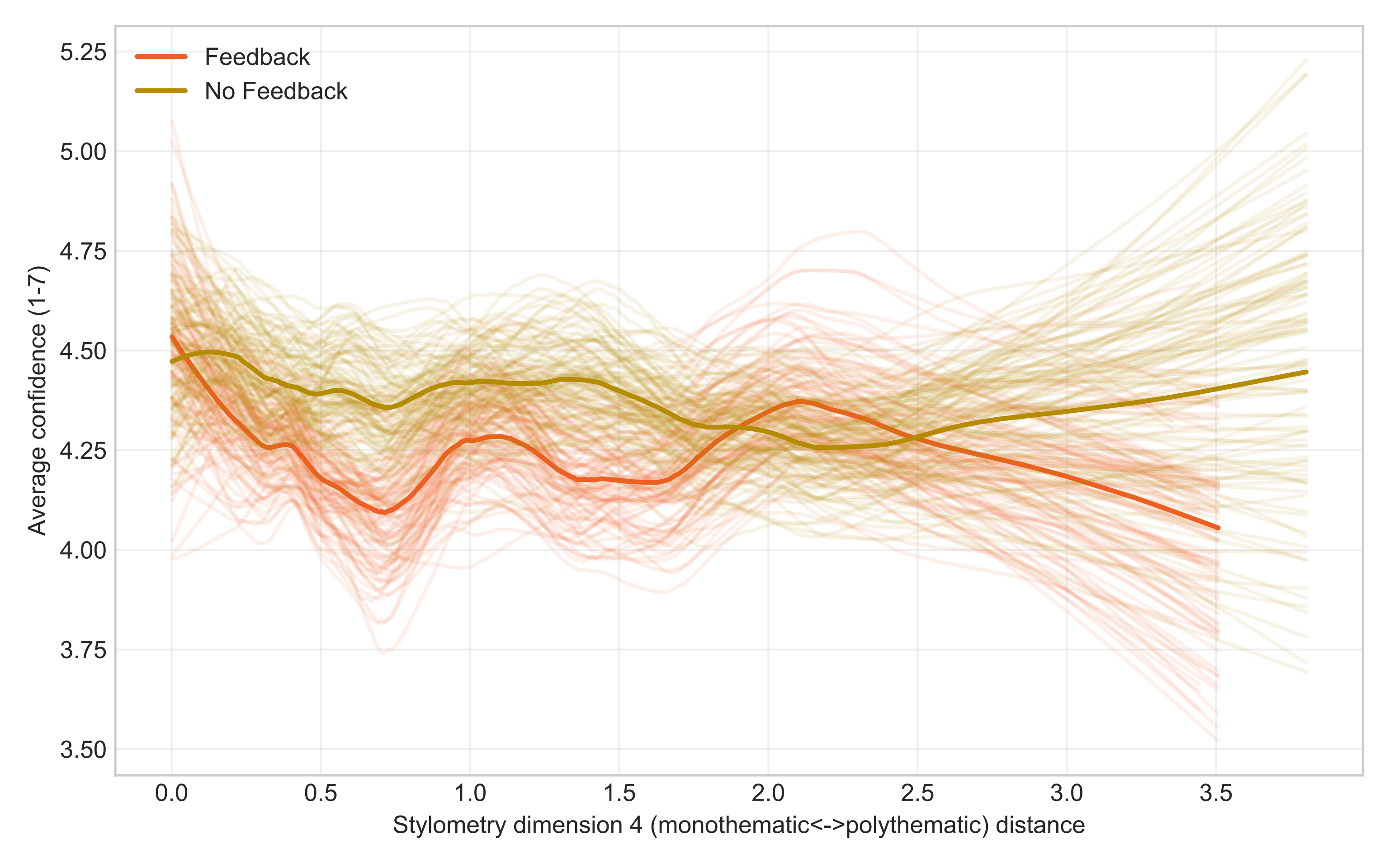

Feedback
No Feedback
Average confidence (1-7)
5.25
5.00
4.75
4.50
4.25
4.00
3.75
3.50
0.0
0.5
1.0
1.5
2.0
2.5
3.0
3.5
Stylometry dimension 4 (monothematic<->polythematic) distance
